# Endovascular Parent Artery Occlusion for Intracranial Aneurysms is a Viable, Cost-Effective Alternative: An Institutional Experience from Northeast India

**DOI:** 10.15388/Amed.2021.28.2.6

**Published:** 2021-08-04

**Authors:** Pranjal Phukan, Kalyan Samra, Donboklang Lynser, Barun Kumar Sharma, Deb Kumar Boruah, Bishwaeet Saikia, Binoy Kumar Singh

**Affiliations:** Department of Radiology & Imaging North Eastern Indira Gandhi Regional Institute of Health & Medical Sciences, Meghalaya, India; Department of Neuroimaging & Interventional Neuroradiology All India Institute of Medical Science, New Delhi; Department of Radiology & Imaging North Eastern Indira Gandhi Regional Institute of Health & Medical Sciences, Meghalaya, India; Department of Radiodiagnosis Sikkim Manipal Institute of Medical Sciences, Sikkim; Department of Radiodiagnosis Tezpur Medical College, Assam; Department of Anatomy North Eastern Indira Gandhi Regional Institute of Health & Medical Sciences, Meghalaya, India; Department of Neurosurgery North Eastern Indira Gandhi Regional Institute of Health & Medical Sciences, Meghalaya, India

**Keywords:** Intracranial Aneurysm, Balloon Occlusion, Endovascular

## Abstract

**Purpose.:**

Endovascular parent artery occlusion (PAO) may be an alternative approach for complex intracranial aneurysm with potentially life-threatening complications. Moreover, the long-term follow-up of the PAO for an intracranial aneurysm is reported sparingly, limited to the case series. It is therefore important to carry out more research on long-term follow-up of the implication of PAO of intracranial aneurysm. The aim of the study was to analyses our experience of PAO for intracranial aneurysms with emphasis on long-term follow-up.

**Materials and Methods.:**

The data of patients treated with PAO for intracranial aneurysms were reviewed. The outcome was evaluated based on aneurysmal occlusion on immediate angiography, follow-up magnetic resonance angiography (MRA), and complications. The modified Rankin score (mRS) was used to evaluate the functional outcome during the last follow-up. The mean, range, and standard deviation were reported for other variables – the patient’s age, number, and percentage.

**Results.:**

Endovascular treatment was performed in 178 patients including PAO in 18 patients. Of these 18 (eighteen) patients, there were 13 dissecting aneurysms, 4 mycotic aneurysms, and one traumatic aneurysm.

10 (ten) patients underwent PAO for proximal intracranial artery aneurysm and 8 (eight) patients for distal cerebral aneurysms. Complete occlusion of the aneurysm was achieved in 16patients (88.89%) and retrograde filling of the aneurysm was seen in 2 (11.11%) patients. One patient had intraprocedural coil migration resulting in a major infarct with an mRS of 2. Another patient (5.56%) had recanalization of the aneurysm and presented with rupture and intracranial hemorrhage with an mRS score of 4. The mRS of the other 16 patients (88.89%) was zero.

**Conclusions.:**

Endovascular PAO for cerebral aneurysms was highly feasible and achieved complete occlusion. The morbidity and mortality rates were at the long-term follow-up also acceptable with negligible complications.

## Abstract (lt)

**Intrakranijinių aneurizmų gydymas taikant endovaskulinę pamatinės arterijos okliuziją – tinkama, pigi alternatyva. Šiaurės Indijos įstaigos patirtis:**

**Santrauka. *****Tikslas.*** Endovaskulinę pamatinės arterijos okliuziją galima taikyti kaip alternatyvą gydant sudėtingą intrakranijinę aneurizmą, galinčią turėti gyvybei pavojingų komplikacijų. Be to, aprašyta tik keletas ilgalaikės kontrolės po intrakranijinių aneurizmų gydymo taikant pamatinės arterijos okliuziją atvejų. Todėl turėtų būti atlikta daugiau ilgalaikės kontrolės po intrakranijinių aneurizmų gydymo taikant pamatinės arterijos okliuziją tyrimų. Tyrimo tikslas – ištirti mūsų patirtį gydant intrakranijines aneurizmas taikant pamatinės arterijos okliuziją, dėmesio skirti ilgalaikei kontrolei.

***Medžiaga ir metodai. ***Peržiūrėti pacientų, gydytų nuo intrakranijinių aneurizmų taikant pamatinės arterijos okliuziją, duomenys. Rezultatai vertinti atsižvelgiant į aneurizmų okliuziją iškart po gydymo atlikus angiografiją, kontrolės metu – magnetinio rezonanso angiografiją (MRA) ir komplikacijas. Modifikuota Rankin skalė (mRS) naudota įvertinti paskutinės kontrolės metu gautus funkcinius rezultatus. Kitiems kintamiesiems nustatyti pacientų amžiaus, kiekio ir procento vidurkis, amplitudė ir standartinis nuokrypis.

***Rezultatai.*** Endovaskulinis gydymas taikytas 178 pacientams, o 18 pacientų atlikta pamatinės arterijos okliuzija. Iš šių 18 (aštuoniolikos) atvejų 13 atvejų buvo atsisluoksniuojančios, 4 atvejais mikotinės aneurizmos ir viena trauminė aneurizma.

10 (dešimčiai) pacientų gydant proksimalinės intrakranijinės arterijos aneurizmą, o 8 (aštuoniems) pacientams – periferinės galvos smegenų aneurizmą, atlikta pamatinės arterijos okliuzija. Visiška aneurizmos okliuzija nustatyta 16 pacientų (88,89 %), 2 pacientams (11,11 %) aneurizma atsinaujino. Vienam pacientui procedūros metu įvyko spiralės migracija, sukėlusi infarktą, vertintiną pagal mRS skalę. Kitam pacientui (5,56 %), atlikus aneurizmos rekanalizaciją, trūko aneurizma ir įvyko intrakranijinis kraujavimas, pagal mRS skalę – 4 balai. Kitų 16 pacientų mRS(88,89 %) balas buvo 0.

***Išvados.*** Gydant galvos smegenų aneurizmas, endovaskulinė pamatinės arterijos okliuzija daugeliu atvejų pasiteisino ir leido pasiekti visišką okliuziją. Vykdant ilgalaikę kontrolę, sergamumo ir mirštamumo rodikliai buvo priimtini, pasitaikė nereikšmingų simptominių išeminių komplikacijų, rekanalizacija.

**Raktažodžiai:** intrakranijinė aneurizma, okliuzija balionėliais, endovaskulinė pamatinės arterijos okliuzija

## Introduction

Endovascular coiling is currently an incredible approach for intracranial aneurysms. Recent approaches like flow diversion make it feasible for complex aneurysms where simple or balloon-assisted coiling is not amenable [1,2]. However, recent pieces of literature demonstrate the high morbidity and mortality using flow-diverter stents [3]. Endovascular parent artery occlusion (PAO) may be an alternative approach in such situations [3, 4], which has been practiced long in the tooth despite the potentially life-threatening complications like delayed ischemia or hemorrhage and de-novo aneurysm formation. Moreover, the long-term follow-up of the PAO for the intracranial aneurysm is reported sparingly limited to the case series. It is therefore important to carry out more research on long-term follow-up of the implication of PAO of intracranial aneurysm. The study aimed to analyze our experience of PAO for intracranial aneurysms with an emphasis on long-term follow-up. 

## Materials and Methods

### Study design and data collection 

This is a retrospective cross-sectional study conducted between April 2013 and December 2020. The study was approved by the Institutional Ethical committee. All the patients with intracranial aneurysm treated with parent artery occlusion were included in the study. The demographic data, clinical presentation, imaging findings, aneurysm location, morphology, and treatment modality of all patients were reviewed. Those patients with inadequate data were excluded from the study.

### Outcome evaluation

The study outcome was evaluated by analyzing the immediate post-procedural complications, and aneurysm occlusion on post-procedure Time-Of-Flight Magnetic Resonance Angiography (TOF MRA) at the last follow-up. The functional outcome was measured by the modified Rankin score (mRS) during the last follow-up. 

### Diagnostic angiography 

A routine cerebral angiography was performed under local anesthesia after taking written informed consent as per our institutional protocol under local anesthesia whenever possible. The procedures were performed using a digital C-Arm (BV Endura Philips Healthcare, Amsterdam, Netherland). A routine six-vessel cerebral angiography with cross compression was performed for all patients. Patients showing good collateral flow on cross compression were only taken up for balloon test occlusion (BTO). All the treatment options – stent-assisted coiling, FD, and PAO – were explained to the patients and their relatives with their risk, benefits, and the approximate cost. Of the 18 patients, 15 patients opted for PAO primarily due to the low cost of the procedure. In another three patients, PAO was performed predicting the possible technical difficulty for mini-stent coiling.

### Proximal artery occlusion 

**Figure 1.**
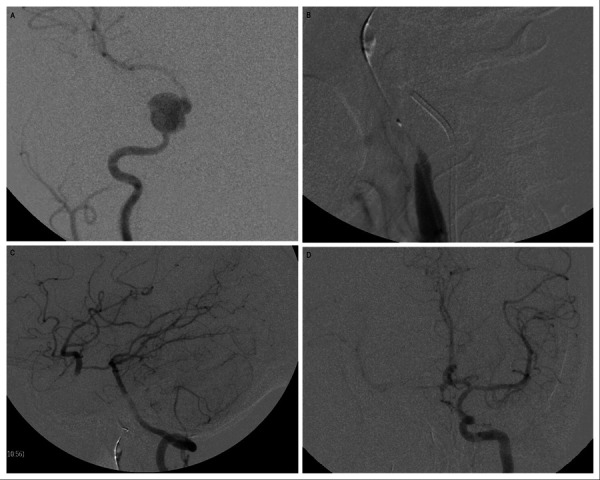
Balloon occlusion test (BTO).

The balloon test occlusion (BTO) was performed before PAO for all patients with proximal intracranial artery aneurysm (Figure1). Under all aseptic precaution and local anesthesia, the common femoral artery was punctured and 80U/kg body weight heparin was injected. A balloon was carefully inflated proximal to the aneurysm and continuous neurologic examination for the motor, sensory, cognition, and cranial nerve functions were performed for 20-min. The PAO was performed only when the patient tolerated the balloon occlusion test without a neurological deficit. During balloon inflation, multiple selective angiograms were performed through the contralateral internal cerebral artery (ICA) and vertebral artery (VA), for demonstration of adequate cross circulation flow.

### Distal cerebral artery occlusion 

The target artery and the aneurysm were selectively negotiated through the guiding catheter in a coaxial fashion. The micro-catheter tip was placed in an adequate position for PAO. Wada test was performed only after occlusion of the parent vessel of the distal cerebral aneurysm. Infusion of low dose heparin according to the weight was instituted for 48 hours to prevent thrombosis. 

A. Angiography of the right ICA reveals a pseudoaneurysm arising from the right cavernous ICA. B. A balloon is inflated at the right petrous ICA proximal to the aneurysm. C. Angiography of the left vertebral artery reveals good cross circulation through Pcom, filling the right ICA circulation. D. Angiography of the opposite ICA reveals no adequate cross circulation through Acom.

### Statistical analysis 

Data were analyzed in Microsoft Excel 2010. The mean, standard deviation, and range for the patient’s age were determined and recorded (Table 1). The number, as well as the percentage of the variables, were determined and recorded (Table 2). 

## Results

The endovascular PAO was performed in 18 patients who were included in the study group with the mean age 42.4 ± 17.7 years (range from 3 months to 75 years) and male to female radio 5:4. All the patients were in good clinical condition at presentation with a full Glasgow Coma Scale (GCS) score at the time of presentation. The clinical presentations were headache in 13 patients (72.22%), cranial nerve palsy in 6 patients (33.33%), weakness in 2 patients (11.1%), seizure in one patient (5.55%), and epistaxis in another one patient (5.55%). On CT/MR imaging subarachnoid hemorrhage (SAH) was detected in 5 patients (27.78%), a mass effect due to aneurysm in 8 patients (44.44%), intraparenchymal hemorrhage in 5 patients (27.78%), and another one patient (5.56%) with a history of road traffic accident one month back had sphenoid sinus hematoma and presented with life-threatening epistaxis (Table 1).

**Table 1.** Showing clinical presentations with imaging findings in patients undergoing PAO and follow-up

Case	Age (years)	Sex	Presentation	CT/MRI	Aneurysmal Type	Location	Size (cm)	Follow-up (Months)	mRS
1	31	F	6th CN palsy	Mass effect	Dissecting	Right Cavernous ICA	4.0	8	0
2	38	M	6th CN palsy	Mass effect	Dissecting	Left Cavernous ICA	4.5	47	0
3	63	F	Headache	Mass effect	Dissecting	Left supraclinoid ICA	1.2	12	0
4	75	M	Headache & CN palsy	Mass effect	Dissecting	Right Cavernous ICA	4.2	3	0
5	31	F	Headache & CN palsy	Mass effect	Dissecting	Right Cavernous ICA	2.0	50	0
6	62	F	Headache	Mass effect	Dissecting	Left Cavernous ICA	1.9	36	0
7	35	F	CN Palsy	Mass effect	Dissecting	Left Cavernous ICA	1.7	42	0
8	23	M	Epistaxis	Hematoma in Sphenoid sinus	Traumatic	Right cavernous ICA	1.5	36	0
9	45	M	Headache	ICH	Mycotic	Left MCA–M4 segment	0.5	12	0
10	56	M	Headache &weakness	ICH	Mycotic	Left MCA–M4 Segment	0.4	42	0
11	0.25	M	Seizure and weakness	ICH	Mycotic	Left MCA–M4 segment	4.0	60	0
12	25	M	Headache	SAH	Dissecting	Right MCA–M 4 segment	0.6	3	0
13	52	M	Headache	ICH with SAH	Dissecting	Right PCA–P2 segment	1.3	36	4
14	25	F	Headache	SAH	Mycotic	Right PCA– parieto-occipital	0.5	4	0
15	45	F	Headache	SAH	Dissecting	Right VA–V4 segment	0.4	60	0
16	45	F	Headache	ICH	Dissecting	Left PICA	0.5	18	0
17	54	F	Headache	SAH	Dissecting	Left supraclinoid ICA	0.9	12	2
18	59	F	Headache with 6th CN palsy	Mass effect	Dissecting	Left cavernous ICA	1.3	12	0

**Abbreviations: **CT – Computed tomography, ICA – Internal carotid artery, ICH – Intracranial hemorrhage, MCA – Middle cerebral artery, MRI – Magnetic resonance imaging, mRS – modified Rankin score at last follow up, PAO – parent artery occlusion, PCA – Posterior cerebral artery, PICA – Posterior inferior cerebellar artery, SAH – Subarachnoid hemorrhage, VA – Vertebral artery

### Aneurysms morphology and location

Out of the total 18aneurysms studied, there were 13dissecting, 4 mycotic aneurysms, and one traumatic aneurysm. Out of the 100% (4/4) of the mycotic and traumatic (1/1) aneurysm, and 38.5% (5/13) of the dissecting aneurysms were ruptured at the time of presentation. The rest 61.5% (8/13) of the dissecting aneurysms were unruptured and presented with mass effect.

Of the 13 dissecting aneurysms, 9 were located in the ICA, one in the M4 segment of the right middle cerebral artery (MCA), one in the P2 segment of the right posterior cerebral artery (PCA), one in the V4 segment of the right vertebral artery, and another one in the left posterior inferior cerebellar artery (PICA). Of the 4 mycotic aneurysms, 3 were located in the M4 segment of the left MCA, and one in the parieto-occipital branch of the right PCA. The post-traumatic aneurysm was located in the right cavernous ICA. The average maximum diameter of the aneurysms was 1.75 cm with 4 giant aneurysms (>25mm in diameter), consisting of 3 dissecting and 1 mycotic aneurysm (Table 1). 

### Embolizing agents 

Out of the ten ICA aneurysms, 0.018” Nester pushable coils (Cook Medical, Bloomington, USA) were used in 4 aneurysms, AMPLATZER Vascular Plug 4 (St. Jude Medical Inc., Minnesota, USA) in one aneurysm, and detachable micro-coils were used in 5 aneurysms (Table 2). PAO of segment proximal to the aneurysm was performed in 7 patients, distal segment aneurysmal sac, as well as proximal segment coiling was done in 3 patients. The right VA aneurysm was managed with detachable micro coils. Out of the 7distal cerebral artery aneurysm occlusions, detachable coils were used in 4 patients and glue in 3 patients (Table 2). 

**Table 2.** Parent artery occlusion of cerebral arteries showing the type of aneurysm, embolization materials used, results and complications

			ICA	MCA	PCA	PICA	VA	TOTAL
1	Type of aneurysm	Dissecting	9	1	1	1	1	13
		Traumatic	1	0	0	0	0	1
		Mycotic	0	3	1	0	0	4
		Pushable coils	4	0	0	0	0	4
2	Embolization	Microcoils	5	2	1	1	1	10
		Amplatzer device	1	0	0	0	0	1
		Glue	0	2	1	0	0	3
3	Result at follow-up	Complete	8	4	2	1	1	16
		Incomplete	2	0	0	0	0	2
4	Complications	No	8	4	2	1	0	15
		Yes	1	0	0	0	1	2

**Abbreviations:** ICA – Internal carotid artery, MCA – Middle carotid artery, PCA – Posterior carotid artery, PICA – Posterior inferior cerebral artery, VA – vertebral artery.

### Immediate outcome 

The procedure was technically successful in all patients with complete occlusion of aneurysm achieved in 16 (88.89%) patients. An immediate post-procedure angiography revealed a retrograde filling of residual aneurysms in 2(11.11%) patients (Table 2). 

### Complications

One patient with a left supraclinoid ICA dissecting aneurysm had acute intraprocedural coil migration during endovascular PAO and resulted in the occlusion of left M1MCA. The migrated coil was successfully retrieved using a TrevoProVue (4-mm-diameter) stent retriever (Stryker). Endovascular PAO was later done using a bigger size 3D coil and a complete occlusion of the aneurysm was achieved. But the patient developed a thromboembolic acute infarct in the left MCA territory and developed weakness of the right upper limb, which later improved on follow-up.

Another patient with a right VA dissecting aneurysm developed a small acute infarct in the right inferior cerebellar peduncle and presented with acute vertigo, and dysarthria in the post-procedure period. The patient was managed conservatively with heparin infusion and anti-platelet drugs, following which there was a complete recovery, and the patient was discharged with no neurological deficits. 

### Long term outcome

The average period of follow-up was 45.4 months. (range from 3 to 72 months) (Table 1). A followup imaging with Magnetic Resonance Angiography (MRA)with Time-Of-Flight (TOF) sequence was done for all patients. One (5.56%) patient, who was treated for right PCA – P2 segment dissecting aneurysm by endovascular occlusion of the parent artery along with the aneurysm, developed recanalization and presented with rebleed, with SAH, and intracranial hemorrhage at 3 years’ follow-up, which was subsequently managed by surgical treatment.

The rest of the 17 (94.44%) patients showed complete occlusion of the aneurysm, with no evidence of regrowth or residual on follow-up.

### Functional outcome 

The modified Rankin score (mRS) at the last follow-up was zero (no residual symptoms) in 16 (88.89%) patients (Table 1). One patient who had intraprocedural coil migration during PAO developed the left MCA territory infarct and her mRS was 2. Another patient who had aneurysm recanalization presented with rebleed, with SAH, and intracranial hemorrhage, at 3 years’ follow-up with mRS score of 4. The overall mortality was nil in this study. 

## Discussion

Newer materials like stent-assisted coiling and FDs are very effective in dealing with complex cases of intracranial aneurysms and are thus gradually replacing conventional methods like PAO [3]. This study reports the experience of a single tertiary institute in the Northeastern part of India with long-term follow-up. 

### Morphology

#### Dissecting aneurysm

In our study, 5 patients had acute dissecting aneurysms. These aneurysms usually present with SAH, and there is a high risk of rebleeding leading to poor prognosis, so urgent treatment is mandatory [5]. Also, the dissecting segment can gradually enlarge resulting in the formation of a serpentine or giant dissecting aneurysm. Although selective endovascular treatment of the aneurismal pouch is usually feasible, the best option remains endovascular PAO at the dissection site along with the aneurismal pouch [3,6]. The concomitant occlusion of the parent artery allows occlusion of the dissected segment of the artery, limits the progression of the dissection, and also prevents rebleeding. To minimize the risk of recanalization from collateral vessel dissecting aneurysms in intradural location should be occluded at the dissecting site, as was done in some of our cases.

In our study 8 patients had chronic dissecting aneurysms, out of these 3 were with partially thrombosed giant aneurysms (>25mm in diameter), and another 3 were with large aneurysms(15– 25mm). Chronic dissecting aneurysms, are usually large or giant lesions and presents with mass effect, and the pathological process involves the arterial wall, and so selective treatment is usually ineffective, leading to high rates of recanalization of the aneurysm [5,6]. Following PAO, the reduction of the thromboses compartment and mass effect is usually observed [7,8].

#### Traumatic aneurysm

In our study group, one patient with a history of road traffic accident one month back developed a traumatic aneurysm in the right cavernous ICA and presented with life-threatening epistaxis. These are mostly pseudoaneurysms and are devoid of a true arterial wall and consist of a hematoma surrounded by a fibrous layer, which may rupture and cause massive epistaxis due to disruption through the sphenoid sinus wall as in our case [9,10]. Even trivial trauma can result in the formation of post-traumatic aneurysms and is associated with high morbidity and mortality as high as 50% with a high incidence of rupture of up to 67%. and so, need urgent management [11,12,13, 14]. Even though surgical clipping is the treatment of choice, as it ensures a definitive exclusion of the aneurysm from the circulation along with evacuation of the hematoma with reduction of the mass effect on the surrounding structures, but it is not always feasible [15]. Moreover, the traumatic intracranial aneurysm has a higher tendency to rupture comparing to a congenital aneurysm during surgical clipping due to a poorly defined wall, absence of neck, and associated arachnoid adhesions in the former. So, surgical clipping without sacrificing the parent artery may be a difficult task with hemorrhagic complications [16]. Therefore, deconstructive PAOand reconstructive flow diversion devices remain viable lifesaving alternatives to surgery. But, the PAO technique doesn’t require anti-platelet therapy which increases the risk of active bleeding as compared to patients treated with flow diversion devices [15,17].

#### Mycotic aneurysms

In our study group, 4 patients had mycotic aneurysms which were all ruptured at the time of presentation. These aneurysms are a rare entity and account for 0.7 to 5.4% of all cerebral aneurysms [18]. Due to the lack of randomized control trials, presently there are no standard management guidelines. Treatment consists of the use of antimicrobial drugs, surgery, endovascular treatment, and/ or a combination of them [19]. Management essentially depends on the aneurysm characteristics, whether it has ruptured or not, and the patient’s overall health status [19,20].

Even though unruptured mycotic aneurysms in patients with high surgical risk, can be conservatively managed with antibiotic therapy, on the other hand, ruptured aneurysms should be urgently treated by surgery or endovascular treatment [20], as in our study group. Surgical clipping of a mycotic aneurysm is technically challenging due to the friable nature of the aneurysm, and absence, or the deformity of its neck [20]. Endovascular treatment strategies of intracranial mycotic aneurysms may include PAO or the use of flow diversion devices or stents [20]. Although there are few case reports on the use of flow diversion devices and stents in the endovascular treatment of mycotic aneurysms, there is only limited knowledge regarding the long-term effectiveness and safety of such devices in the setting of active infection [21,22,23]. Moreover, another major drawback of the use of flow diversion devices and stents in the endovascular treatment of mycotic aneurysm is the use of antiplatelet agents [23], which can be critical in cases of a ruptured aneurysm, and the risk of stent infection, a dreaded complication. In this regard, PAO still acts as an effective and viable alternative. 

Although some risks related to BTO have been reported [24], but still, it is a preliminary and determinant step in planning any endovascular treatment. In those patients in whom clinical conditions prevent neurological evaluation, even an exclusively angiographic occlusion test is usually sufficient in planning further endovascular treatment. In cases of peripherally located aneurysms, distal branches of the anterior, middle, and posterior cerebral arteries and also on cerebellar arteries evaluation of the collateral leptomeningeal circulation sometimes be difficult, but usually feasible and needs careful evaluation of the cerebral angiograms [5]. Moreover, despite the good angiographic collaterals distal to the site of occlusion, complications may occur [25]. On the other hand, even without previously identified collateral circulation, good outcomes can be observed [26].

### Distal artery occlusion

Distal cerebellar artery aneurysms are rare and constitute only 0.6% of all intracranial aneurysms [27]. Amidst the controversy between selective embolization and PAO, justifiable reports on PAO for distal intracranial aneurysms subsist [28]. No prior provocative test was performed in our study in all the cases of distal artery aneurysms, as it was seen that there was a presence of pre-existing collaterals. In a study of PAO where 12 distal cerebral aneurysms were treated with detachable coils, no new-onset neurologic deficits were reported [29]. In another study, the complete occlusion rate of the low profile mini stent-assisted aneurysmal coiling was 75% out of 80 patients on immediate post-procedural angiography, increase filling status was found in 85.7% out of 77 patients who came for follow-up angiography, 5.2% aneurysms needed retreatment, complications were encountered in 11.3% of patients and permanent disability was found in 3.8% of patients [30].

### Proximal artery occlusion 

Even if certain carotid and VA aneurysm can be treated effectively by endovascular PAO [31], posterior circulation fusiform/dissecting aneurysm has a rupture rate as high as 71.4% [32]. The desired outcome of the PAO of a fusiform/dissecting aneurysm can be achieved by inducing thrombosis followed by involution [3]. There are limited studies on patient outcomes after PAO in a vertebrabasilar aneurysm. Kai et al. successfully performed PAO of 11 VA dissecting aneurysms using coils without any major complications [33]. The endovascular PAO of the ICA can be performed effectively with detachable balloons and more recently with micro coils [34]. Kuramoto et al reported successful ICA occlusion of 34 aneurysms of 33 patients with periprocedural ischemia in four patients (12%) [35]. A recent study of PAO for treatment of 17 giant CCA aneurysms, revealed complete occlusion in all patients with periprocedural infarct in 5.9% of cases and there was no mortality [36]. Out of the 8 ICA aneurysms in our study, we used pushable or micro coils in 7 patients and Amplatzer vascular plugin 1 patient. Out of 10 ICA aneurysms, 2 patients had a retrograde residual filling of aneurysms immediately after occlusion of the ICA, which were eventually thrombosed on follow-up indicating a very slow rate of the retrograde flow. 

### Outcome

#### Complications

**Figure 2.**
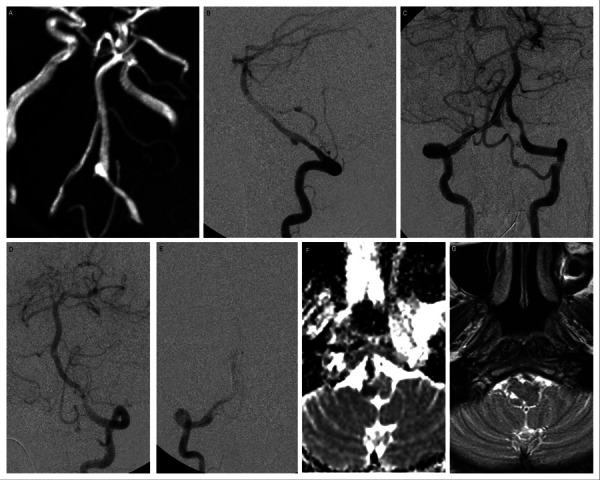
PAO of vertebral artery dissecting aneurysm.

We performed PAO of the VA dissecting aneurysm in one patient (Figure 2). In post-procedure, there was complete occlusion of the parent artery without filling of the aneurysm. However, the patient developed acute vertigo and dysarthria during the first 24 hours after the procedure. The MRI showed a small acute infarct in the right inferior cerebellar peduncle. The patient was then treated conservatively with corticosteroids and heparinization followed by oral aspirin. The mean blood pressure was kept on the higher side (mean BP= 100 mm Hg). The patient completely recovered on follow-up. As it was reported, insufficient crossflow or thrombo-embolic phenomenon from the occlusion site may lead to late-onset ischemic events [37], so we can presume a transient thromboembolic episode in a small perforating artery for such presentation in our case.

45 years old female presented with headache and SAH. (A) TOF MR angiogram flipped image shows a gradual narrowing of the right V4 vertebral artery with a fusiform dilatation. (B) The lateral projection of the right vertebral artery angiogram reveals a dissecting aneurysm arising from the V4 segment. (C) Bilateral vertebral artery angiogram, with the microcatheter placed for PAO. The left PICA was supplying the right PICA territory. (D and E) Post-coiling check– left VA angiogram shows complete obliteration of the aneurysm. In the immediate post-procedural period, the patient developed vertigo and dysarthria. (F)MRI performed 2 days after the procedure, DWI showed a small infarct at the right inferior cerebellar peduncle. (G) Follow-up MRI T2WI showed a small area of gliosis in the same area. The patient had full recovery during her hospital stay and was discharged with no neurological deficits.

In our series, one patient had an acute intraprocedural coil migration during the endovascular PAO of the left supraclinoid ICA dissecting aneurysm causing occlusion of left M1MCA. Even though the migrated coil was successfully retrieved by stent retriever and endovascular PAO was later done using a bigger size 3D coil with complete occlusion of the aneurysm, the patient developed acute thromboembolic infarct in the left MCA territory, and presented with weakness of the right upper limb. Out of the total 178 aneurysms treated by endovascular means during the study period, this was the only case of coil migration (0.56%). Coil migration is a rare complication associated with endovascular coiling with a reported incidence of 0.3–6% in the literature, which may result in catastrophic consequences if not effectively managed on time [38]. Abdalkader et al. reported the use of smaller, undersized coils relative to the maximal aneurysm size as one of the factors associated with coil migration, as was also seen in our case [38]. However, in our patient, the safe and timely retrieval of the migrated coil prevented the expansion of the infarct core, thereby limiting the patient’s disability, with an mRS score of 2 on follow-up. Even though coil retrieval is a high-risk procedure, which may result in hemorrhage and/or thromboembolism, it should be considered as the primary modality using stent retrievers especially in intraprocedural migration, with the proximal location of the migrated coil, and/or associated with vessel occlusion [38]. 

#### Recanalization

In our series, we observed recanalization in one patient treated for right PCA–P2 segment dissecting aneurysm by PAO along with the aneurysm, who later presented with rebleed, with SAH, and intracranial hemorrhage, at 3 years follow- up. Qin X et al. in their series of 59 PCA aneurysms treated by endovascular approach reported recanalization in 2 patients who were treated by PAO with occlusion of the aneurysm and postulated the following causes. Firstly, inadequate packing during initial coiling might contribute to coil compaction and recanalization. Secondly, during the initial treatment, a microcatheter might be placed into the false lumen of the dissecting aneurysm thereby occluding the lumen. Later with gradual healing, the compressed true lumen may expand leading to recanalization [39]. 

The limitations of this study include its retrospective design with a small sample size and followup over a limited period. The study group was also heterogeneous for the location and morphology of the aneurysms which might act as a confounding factor affecting the outcome. Although there was a favourable outcome for PAO in intracranial aneurysms in this study, it is a prerequisite to analyzing the morphology, location of an aneurysm, and existing collaterals before the procedure. Moreover, a risk of long-term complications like aneurysm recanalization, ischemia to the ipsilateral hemisphere, the formation of a new aneurysm on the contralateral side, or increase in the size of any preexisting contralateral aneurysm and intracranial hemorrhage exists, which makes PAO a grey area for both the patient and interventionist and demands long term follow-up. A more comprehensive and detailed study with bigger sample size and a longer follow-up period might reflect a better picture for the best possible treatment approaches. 

## Conclusion

PAO for cerebral aneurysms was highly feasible and achieved complete occlusion. The morbidity and mortality rates were at the long-term follow-up also acceptable, complications like symptomatic ischemia and recanalization of the aneurysms are negligible. It is a safe, viable, and cost-effective alternative for selective intracranial aneurysms which comes up with a huge cost advantage compared to the newer procedures without compromising the outcome.
